# ICU environment as a reservoir of KPC-ST307-*Klebsiella pneumoniae* high-risk clone resistant to ceftazidime-avibactam

**DOI:** 10.1038/s41598-025-14987-w

**Published:** 2025-08-12

**Authors:** Marta Hernández-García, Marta Nieto-Torres, Natalia Guerra-Pinto, Juan Antonio Castillo-Polo, Javier Saez de la Fuente, Malkoa Michelena, Manuel Ponce-Alonso, Cruz Soriano-Cuesta, Cristina Díaz-Agero, Rafael Cantón, Teresa M. Coque, Patricia Ruiz-Garbajosa

**Affiliations:** 1 Servicio de Microbiología, Hospital Universitario Ramón y Cajal and Instituto Ramón y Cajal de Investigación Sanitaria (IRYCIS), Madrid, Spain; 2 Present Address: CIBER de Enfermedades Infecciosas (CIBERINFEC), Instituto Salud Carlos III (ISCIII), Madrid, Spain; 3 Servicio de Farmacia, Hospital Universitario Ramón y Cajal and Instituto Ramón y Cajal de Investigación Sanitaria (IRYCIS), Madrid, Spain; 4 Unidad de Cuidados Intensivos, Hospital Ramón y Cajal, Madrid, Spain; 5 Servicio de Medicina Preventiva y Salud Publica, Hospital Universitario Ramon y Cajal and Instituto Ramón y Cajal de Investigación Sanitaria (IRYCIS), Madrid, Spain

**Keywords:** CAZ/AVI-resistance, KPC variants, ST307* K. pneumoniae*, Environmental reservoirs, ICU, COVID-19 pandemic, Microbiology, Molecular biology

## Abstract

We characterized all ceftazidime-avibactam-resistant KPC-producing *K. pneumoniae* (KPC-Kp) isolates recovered from both patients and environmental samples at the ICU of our hospital in 2020, during the COVID-19 pandemic initiation. Antimicrobial susceptibility testing (Sensititre-EUMDROXF; disk-diffusion) and WGS analysis (Illumina-Novaseq/Miseq; Oxford Nanopore^®^-MinION) were performed. Ten patients (16% of ICU patients) were colonized/infected by a ceftazidime-avibactam-resistant KPC-Kp isolate (March-December), six of them during/after treatment with ceftazidime-avibactam. Two ceftazidime-avibactam-resistant KPC-Kp were also recovered from two ICU sinks (July-September). All isolates belonged to ST307 clone and had identical resistance gene content. Six KPC-variants were detected in patient isolates (KPC-62, KPC-92, KPC-150, KPC-66, KPC-53, KPC-46). KPC-92 and KPC-66 variants were also detected in sink isolates. Regardless of the origin (patients or sinks), KPC-92-, KPC-150 and KPC-62-Kp isolates combining altered porin proteins also exhibited increased/resistant MIC values to cefiderocol, cefepime-taniborbactam, aztreonam-avibactam, meropenem-vaborbactam and/or imipenem-relebactam. A cgMLST analysis demonstrated the clonal spread of KPC-ST307-Kp within the ICU, between patients and the hospital environment. Clustering was observed mainly by KPC variants (KPC-92, KPC-62). A variant calling analysis and plasmid characterization showed possible transmission between patients and sinks. Our results suggest that the patient care environment likely contributed to persistence and spread of last-line antibiotics-resistant KPC-Kp within the ICU during the COVID-19 pandemic.

**Supplementary Information:**

The online version contains supplementary material available at 10.1038/s41598-025-14987-w.

## Introduction

*Klebsiella pneumoniae* is a significant nosocomial pathogen, particularly in intensive care units (ICUs), where infections caused by carbapenem-resistant strains have become a critical concern. These strains are often associated with the production of carbapenemases, such as *Klebsiella pneumoniae* carbapenemase (KPC), which confer resistance to a wide range of β-lactam antibiotics, including carbapenems, and involve defects of outer membrane porins that reduce permeability of β-lactams^1,2^. The emergence and spread of KPC-producing *K. pneumoniae* (KPC-Kp) has posed a serious challenge to infection control practices in healthcare settings, especially due to their ability to colonize patients, cause outbreaks and persist in hospital settings^3,4^. Currently, high-risk clones (HiRCs), such as ST307, play a major role in the spread of resistance, as they possess genetic characteristics that may provide an advantage in adaptation to the human host, but also to the hospital environment^5,6^.

Ceftazidime-avibactam is a combination of a third-generation cephalosporin with a β-lactamase inhibitor, that was introduced in 2015 as a novel therapeutic option for treating infections caused by carbapenem-resistant Enterobacterales, including KPC-Kp^7^. Despite its success, reports of resistance to ceftazidime-avibactam have emerged associated with mutations in the KPC enzyme and changes in outer membrane porins, particularly among KPC-Kp strains^8–10^.

Between 2019 and 2020, KPC-3 was the predominant carbapenemase among all *K. pneumoniae* clinical isolates collected in our hospital^11^. In 2020, the COVID-19 pandemic had a major impact on epidemiology, antimicrobial prescribing and resistance to novel antimicrobials in our institution, especially in patients with severe infections and admitted to critical units^12,13^. In the present study, we aimed to investigate the occurrence and characteristics of ceftazidime-avibactam resistant KPC-Kp isolates recovered from patients and sinks of a large ICU ward in our hospital during the COVID-19 early pandemic period (year 2020). We conducted microbiological and molecular analyses to identify resistance mechanisms to ceftazidime-avibactam and to assess the genetic relatedness of clinical and environmental KPC-Kp isolates.

## Results

### Bacterial isolates, patient`s data and ceftazidime-avibactam resistance

**Fig. 1:**
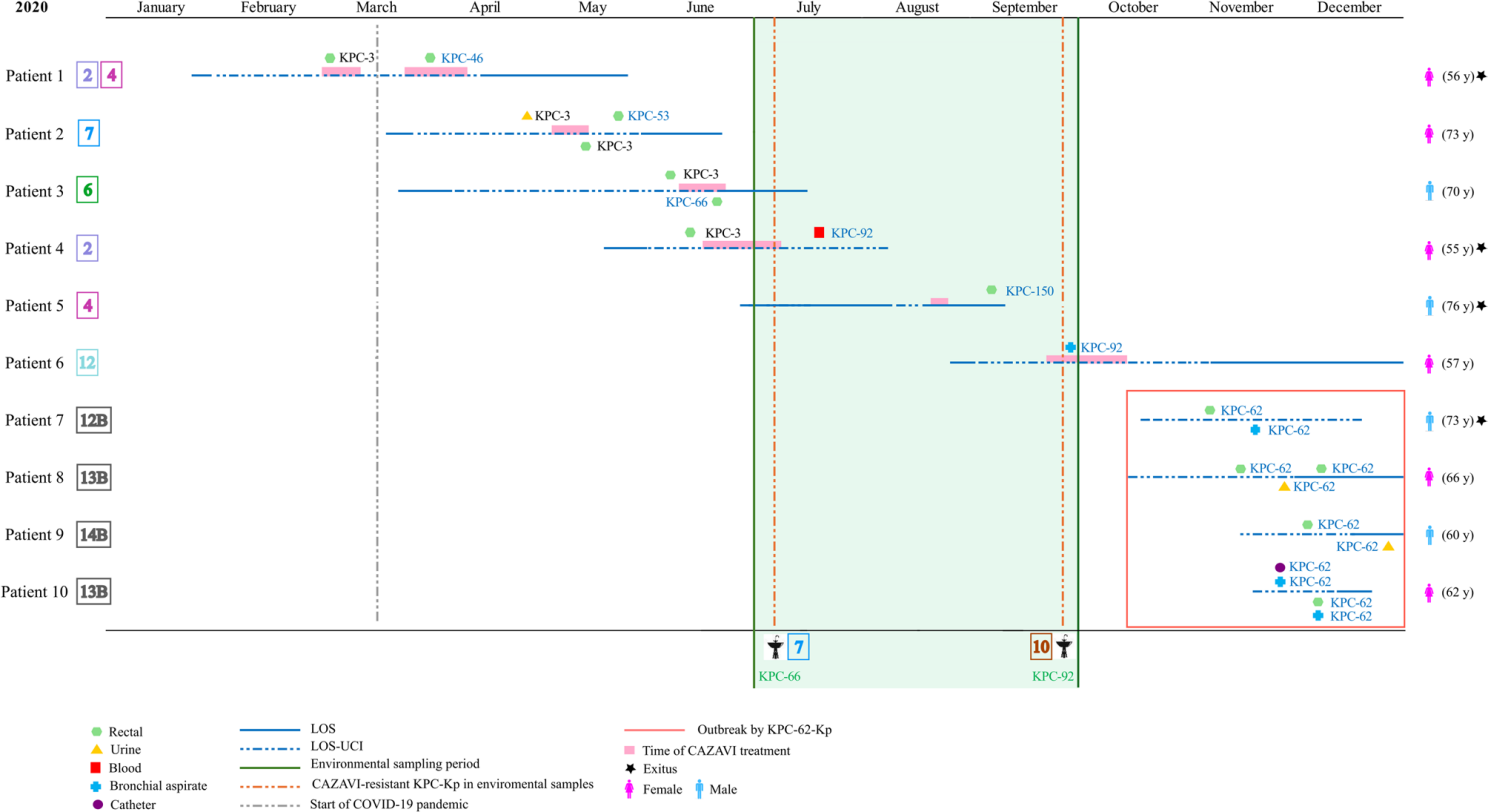
Timeline of events in the ICU of our hospital during the year 2020. KPC-Kp (susceptible and resistant to ceftazidime-avibactam) recovered from patient between January and December are represented horizontally. KPC-Kp (resistant to ceftazidime-avibactam) recovered from environmental samples collected between July and September are represented in vertical (green zone). Length of stay (LOS) and LOS at the ICU of patients are indicated with a blue line and a dotted blue line, respectively. The ICU room where the patients stayed and where the environmental samples were collected is indicated by the framed number (on the left and below, respectively). Time of treatment with ceftazidime-avibactam (dose 2/0.5 g /8 h iv) is represented with a pink bar. The type of clinical sample from which KPC-Kp was isolated is represented by colored symbols (rectal, green hexagon; urine, yellow triangle; blood, red square; bronchial aspirate, blue cross; and catheter, purple circle). Patients involved in the KPC-62-Kp outbreak are framed in the red box.

During 2020, 1,110 carbapenemase-producing *K. pneumoniae* isolates (390 clinical and 720 from rectal samples) were detected in 292 patients admitted to our hospital. Up to 64 of these patients (64/292, 21.9%) were admitted at the UCI and in 41 of them (41/64, 64.1%), a KPC-Kp isolate was detected. KPC-Kp isolates resistant or with decreased susceptibility to ceftazidime-avibactam (MIC_CZA_: 8->16 mg/L) were detected in 10 ICU patients (10/64, 15.6%) between March and December 2020 (Table 1; Fig. 1). These isolates were recovered from rectal colonization samples in nine patients (9/10, 90%) and from clinical samples (three bronchial aspirates, two urines, one catheter and one blood) in five patients (5/10, 50%). The median length of stay (LOS) of these patients was 89 days (range, 26 to 129 days), and the median LOS at the ICU was 61 days (range, 2 to 76 days). Seven of these 10 patients had COVID-19 infection during their ICU stay. Infection control measures were applied to both patients and surfaces immediately after identification of each new case of colonization and/or infection.

**Table 1:** Epidemiological data and antimicrobial susceptibility results of KPC-Kp resistant to ceftazidime-avibactam detected in the ICU of our hospital in both patients (year 2020) and environmental (July-September 2020) samples.

Patients / Sinks (ICU Room)	Origin	Length of stay ay ICU (days)	CZA exposure duration (time of culture)	MIC (mg/L)																			KPC allele
				PT4		AZT	FEP	C/T	CZA	IMI	IMR	MER	MEV	FDC	AZA	FTB	TGC	ERV	COL	FOS	AMK	TOB	
Patient 1 (2, 4)	Rectal	121 (75) days	14 (1) days	> 32/4		> 32	> 16	> 8/4	2/4	16	0.25	8	≤ 0.06/8	0.5	≤ 0.25/4	0.25/4	≤ 0.5	0.5	> 16	≤ 16	≤2	> 4	KPC-3
	Rectal		14 (6) days	> 32/4		> 32	16	> 8/4	8/4	≤ 1	0.25	≤ 0.12	≤ 0.06/8	1	0.5/4	1/4	≤ 0.5	0.25	8	≤ 16	16	> 4	KPC-46
Patient 2 (7)	Urine	91 (61) days	0	> 32/4		> 32	> 16	> 8/4	1/4	> 8	0.5	> 16	≤ 0.06/8	0.5	≤ 0.25/4	0.5/4	≤ 0.5	> 0.5	16	32	8	> 4	KPC-3
	Rectal		6 (6) days	> 32/4		> 32	> 16	> 8/4	2/4	> 8	0.5	> 16	≤ 0.06/8	0.5	≤ 0.25/4	0.5/4	≤ 0.5	0.5	≤ 0.5	≤ 16	4	> 4	KPC-3
	Rectal		6 (14) days	> 32/4		> 32	> 16	> 8/4	16/4	≤ 1	0.25/4	≤ 0.12	≤ 0.06/8	2	≤ 0.25/4	0.5/4	1	> 0.5	> 16	32	4	> 4	KPC-53
Patient 3 (6)	Rectal	109 (76) days	0	> 32/4		> 32	> 16	> 8/4	2/4	> 8	0.25/4	> 16	≤ 0.06/8	1	≤ 0.25/4	0.5/4	≤ 0.5	> 0.5	> 16	≤ 16	4	> 4	KPC-3
	Rectal		11 (8) days	32/4		32	8	8/4	8/4	≤ 1	0.25/4	≤ 0.12	≤ 0.06/8	2	≤ 0.25/4	0.5/4	1	> 0.5	16	≤ 16	≤2	> 4	KPC-66
Patient 4 (2)	Rectal	129 (67) days	0	> 32/4		> 32	> 16	> 8/4	2/4	8	0.25/4	16	≤ 0.06/8	1	≤ 0.25/4	0.25–0.5	≤ 0.5	0.5	≤ 0.5	≤ 16	4	> 4	KPC-3
	Blood		15 (24) days	32/4		> 32	8	8/4	8/4	≤ 1	0.25/4	≤ 0.12	≤ 0.06/8	2	≤ 0.25/4	0.5/4	1	0.5	≤ 0.5	≤ 16	≤2	> 4	KPC-92
Patient 5 (4)	Rectal	79 (73) days	2 (16) days	> 32/4		> 32	> 16	> 8/4	> 16/4	2	2/4	16	4/8	8	2/4	16/4	1	> 0.5	16	> 64	4	> 4	KPC-150
Patient 6 (12 A)	Rectal	72 (2) days	14 (5) days	> 32/4		> 32	> 16	> 8/4	8/4	≤ 1	0.25/4	≤ 0.12	≤ 0.06/8	0.5	≤ 0.25/4	0.25/4	1	>’0.5	≤ 0.5	≤ 16	4	> 4	KPC-92
Patient 7 (12B)	Rectal	58 (56) days	No treated	> 32/4		> 32	> 16	> 8/4	> 16/4	4	1/4	4	1/8	4	1/4	8/4	≤ 0.5	0.5	≤ 0.5	> 64	4	> 4	KPC-62
	Bronchial aspirate			> 32/4		> 32	> 16	> 8/4	16/4	4	2/4	8	2/8	8	1/4	8/4	≤ 0.5	0.5	≤ 0.5	> 64	4	> 4	KPC-62
Patient 8 (13B)	Rectal	89 (32) days	No treated	> 32/4		> 32	> 16	> 8/4	16/4	8	1/4	4	1/8	8	1/4	8/4	≤ 0.5	0.5	≤ 0.5	> 64	4	> 4	KPC-62
	Urine			> 32/4		> 32	> 16	> 8/4	> 16/4	8	0.25/4	8	0.25/8	> 8	1/4	8/4	≤ 0.5	0.5	≤ 0.5	> 64	8	> 4	KPC-62
	Rectal			> 32/4		> 32	> 16	> 8/4	> 16/4	8	0.25/4	8	0.5/8	8	1/4	8/4	≤ 0.5	0.5	≤ 0.5	> 64	8	> 4	KPC-62
Patient 9 (14B)	Rectal	49 (29) days	No treated	> 32/4		> 32	> 16	> 8/4	> 16/4	4	2/4	8	2/8	4	1/4	8/4	≤ 0.5	0.5	≤ 0.5	> 64	4	> 4	KPC-62
	Urine			> 32/4		> 32	> 16	> 8/4	> 16/4	4	1/4	8	1/8	4	1/4	8/4	≤ 0.5	0.5	≤ 0.5	> 64	8	> 4	KPC-62
Patient 10 (13B)	Catheter	26 (19) days	No treated	> 32/4		> 32	> 16	> 8/4	> 16/4	8	0.25/4	8	0.25/8	8	1/4	8/4	≤ 0.5	0.5	≤ 0.5	> 64	4	> 4	KPC-62
	Bronchial aspirate			> 32/4		> 32	> 16	> 8/4	> 16/4	8	2/4	8	2/8	4	1/4	8/4	≤ 0.5	0.5	≤ 0.5	> 64	4	> 4	KPC-62
	Bronchial aspirate			> 32/4		> 32	> 16	> 8/4	> 16/4	8	1/4	8	2/8	> 8	1/4	8/4	≤ 0.5	0.5	≤ 0.5	> 64	4	> 4	KPC-62
	Rectal			> 32/4		> 32	> 16	> 8/4	> 16/4	4	1/4	8	2/8	8	1/4	8/4	≤ 0.5	0.5	≤ 0.5	> 64	4	> 4	KPC-62
Sink 1 (7)	Drain	-	-	> 32/4		> 32	> 16	> 8/4	16/4	2	0.25/4	≤ 0.12	≤ 0.06/8	1	≤ 0.25/4	0.25/4	1	>’0.5	16	≤ 16	4	> 4	KPC-66
Sink 2 (10)	Siphon	-	-	> 32/4		> 32	> 16	> 8/4	16/4	2	2/4	8	4/8	4	0.5/4	16/4	1	>’0.5	≤ 0.5	> 64	32	> 4	KPC-92

*PT4*  piperacillin-tazobactam,* AZT* aztreonam,* FEP * cefepime,* C/T * ceftolozane-tazobactam,* CZA * ceftazidime-avibactam,*IMI  * imipenem,* IMR* imipenem-relebactam,* MER*  meropenem,* MEV * meropenem-vaborbactam,* FDC*  cefiderocol,* AZA * aztreonam-avibactam,* FTB* cefepime-taniborbactam,* TGC * tigecycline, *ERV * eravacycline,* COL* colistin,* FOS * Fosfosmycin,* AMK*  amikacin,* TOB*  tobramycin.

Notably, ceftazidime-avibactam consumption in the ICU increased in 2020, with a DDD/100 patient-days value rising from 0.39 in 2019 to 0.95 in 2020, resulting in a year-on-year variation of 1.41. Among the 64 ICU-admitted patients in 2020, 46 (71.9%) received ceftazidime-avibactam during their stay. In six of these patients (6/46, 13%), ceftazidime-avibactam-resistant KPC-Kp isolates were recovered during or after treatment with ceftazidime-avibactam, and in four of them, a previous KPC-3-Kp isolate susceptible to ceftazidime-avibactam (MIC_CZA_: 1–2 mg/L) had been isolated before starting treatment. In these patients, the median days of treatment with ceftazidime-avibactam until the detection of a ceftazidime-avibactam resistant KPC-Kp isolate was 11 days (range, 5 to 24 days) (Table 1). The four patients with a ceftazidime-avibactam resistant KPC-Kp who had not been treated with ceftazidime-avibactam were admitted to the ICU between November and December and were part of a previously characterized outbreak of KPC-62-*K. pneumoniae* resistant to both ceftazidime-avibactam and cefiderocol^14^.

Between July-2020 and September-2020, 350 environmental samples were also collected from abiotic surfaces, including sinks (drain, siphon and basin) and rooms surfaces (control-breather screen and bed rails), from the ICU of our Hospital as part of the MicroCarbaFlux Project (Ref. CC23140547)^15^. Up to 407 *K. pneumoniae* isolates with a different colony morphotype and antibiotic susceptibility phenotype were recovered. During this screening, two ceftazidime-avibactam resistant KPC-Kp isolates were detected from two samples collected in a drain and a siphon from two sinks in two different ICU rooms (Table 1; Fig. 1).

### Description of cases and antimicrobial susceptibility testing

 Ceftazidime-avibactam KPC-Kp isolates were recovered from ten patients admitted to 8 different ICU rooms (Fig. 1). Up to six KPC variants were detected in isolates from patients, some of them in more than one patient (4 KPC-62, 2 KPC-92, 1 KPC-150, 1 KPC-66, 1 KPC-53 and 1 KPC-46). Two of these KPC variants (KPC-92 and KPC-66) were also identified in KPC-Kp isolates from sink samples. A KPC-66-Kp was collected from a drain of a sink from room 7, 44 days after the collection of a clinical KPC-53-Kp isolate from patient 2 in the same room, and 17 days after the collection of a KPC-66-Kp isolate from patient 3, in the adjacent room (room 6). A KPC-92-Kp was recovered from a siphon of a sink in room 10, 77 days after the isolation of a clinical KPC-92-Kp in patient 4 (room 2), and one day before the detection of another KPC-92-Kp in patient 6 (room 12) (Fig. 1).

Ceftazidime-avibactam resistant isolates that carried variants KPC-66 (from both patient and sink), KPC-92 (from patient), KPC-46 and KPC-53, showed collateral sensitivity to carbapenems (MIC_IMP_ ≤ 1 mg/L; MIC_MER_ ≤ 0.12 mg/L). Isolates carrying KPC-150, KPC-62 and KPC-92 (from the sink), displayed the highest MIC values to ceftazidime-avibactam (MIC_CZA_: 16->16 mg/L), decreased susceptibility to carbapenems (MIC_IMP_: 2–8 mg/L; MIC_MER_: 4–16 mg/L), and were resistant to cefiderocol (MIC_FDC_: 4->8 mg/L). Moreover, these isolates also showed increased MIC values or were eventually associated with resistance to other novel beta-lactam/beta-lactamase inhibitor combinations (Table 1).

### Molecular typing and resistance gene content

All ceftazidime-avibactam resistant KPC-Kp isolates, from both patients and sinks, belonged to the HiRC ST307. An identical acquired-resistance gene content affecting different antimicrobial groups was found: β-lactams (*bla*_CTX−M−15_, *bla*_KPC_, *bla*_OXA−1_, *bla*_OXA−9−*like*_ [W112X], *bla*_SHV−11_, *bla*_TEM−1 A_, *bla*_TEM−1B_), aminoglycosides [*aac(3)-IIa*, *aac(6’)-Ib-cr*, aac(6’)-Ib-cr, *aph(3’’)-Ib*, *aph(6)-*Id], quinolones (*oqxA5*,* oqxB19*, *qnrB1*), sulfonamides (*sul2*), fosfomycin (*fosA6*), chloramphenicol (*catB3*) and trimethoprim (*dfrA14*). Disinfectant resistant genes were not found.

Porin proteins suggested to be unfunctional were present in the clinical KPC-150-ST307-Kp (truncated OmpK36 and OmpK35-Q350X) and the sink KPC-92-ST307-Kp (OmpK36-Ins179_180GE, Ins287_293EVVAQYQ) isolates, both resistant to cefiderocol and cefepime-taniborbactam and with increased MIC values to other novel antimicrobial combinations. In addition, all cefiderocol resistant KPC-62-ST307-Kp isolates carried mutated OmpA (S138F) and OmpR/EnvZ (Y52C, Q103L) genes. Mutations in other genes previously found to be involved in cefiderocol resistance were not detected. The remaining KPC-ST307-Kp isolates, susceptible to carbapenems, cefiderocol and other novel combinations showed intact and identical porin proteins (Table 1). Additionally, all KPC-ST307-Kp isolates carried the conserved ParC-S80I and GyrA-S83I fluoroquinolone resistance-associated mutations.

### cgMLST analysis

**Fig. 2:**
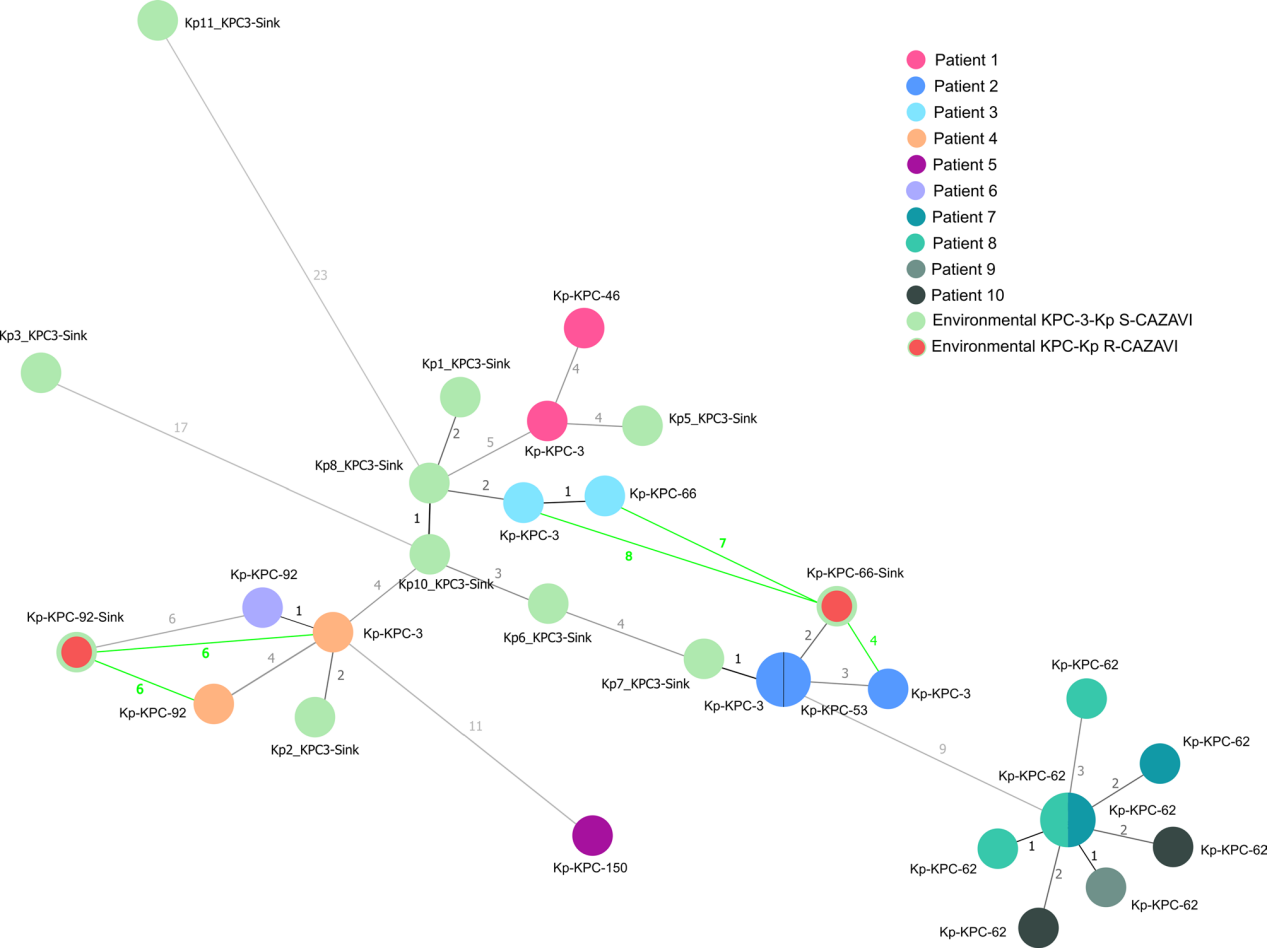
Minimum spanning tree (MST) constructed based on the cgMLST of all KPC-Kp (susceptible and resistant to ceftazidime-avibactam) collected from patients (year 2020) and from environmental samples (July-September 2020) at the ICU of our hospital. KPC-Kp isolates collected from environmental samples are indicated with green circles (totally green, susceptible to ceftazidime-avibactam; red-filled, resistant to ceftazidime-avibactam). The NLV (N locus variant) graph (green line) was calculated between patients and environmental isolates producing the same KPC variant (KPC-92-Kp and KPC-66-Kp).

A cgMLST scheme was created and validated using the ST307-*K. pneumoniae* NCTN00000000 as the reference genome. Nine KPC-3-ST307-Kp isolates with susceptible MIC values to ceftazidime-avibactam recovered during the environmental sampling were also included in this analysis. Of a total of 5,217 loci searched, 4,655 loci were present in the genome of all analyzed KPC-ST307-Kp isolates, which diverged in distance from 0 to 23 loci. The minimum spanning tree showed that isolates from the same patient clustered together (range 0–4 loci), irrespective of whether they were susceptible (KPC-3 producers) or resistant to ceftazidime-avibactam (other KPC producers). Overall, most of the KPC-3-ST307-Kp sink isolates showed a high genetic relationship with patient isolates (≤ 10 loci). Patients were grouped according to the KPC variant they produced (KPC-92 or KPC-62), regardless of the ICU room to which they were admitted. All KPC-62-ST307-Kp resistant to both ceftazidime-avibactam and cefiderocol involved in the outbreak were grouped together and showed a low genetic distance (range 0–3 loci) (Fig. 2).

The KPC-92-ST307-Kp sink isolate (recovered in room 10) was genetically related to clinical KPC-3- and KPC-92-ST307-Kp isolates from patients 6 and 4 (6 loci of difference from each other), despite being collected in different ICU rooms (room 12A and room 2, respectively). However, the KPC-66-ST307-Kp sink isolate was more genetically similar to clinical isolates recovered from patient 7 (KPC-3- and KPC-53-ST307-Kp; range, 2–4 loci), who had been admitted to the same ICU room where the environmental sample was collected (room 7), than with the clinical isolates KPC-3- and KPC-66-ST307-Kp from patient 3 (room 6) (range, 7–8 loci) (Fig. 2).

### Variant calling analysis and plasmid characterization

 A cgSNP based phylogenetic tree was constructed with all KPC-ST307-Kp isolates using the ST307-*K. pneumoniae* NCTN00000000 as the reference genome (Figure S1). A phylogenetic analysis of the KPC-92-Kp sink isolate (used as reference genome) and the KPC-3- and KPC-92-ST307-Kp clinical isolates from patients 4 and 6, respectively, was also performed using hybrid genome assemblies from the combination of short- and long-reads. The core genome ranged from 5,501,653 pb to 5,511,375pb with an evolutionary distance of 5–8 SNPs between these three isolates (Figure S2, Table S1).

Hybrid assemblies’ analysis also revealed that *bla*_KPC−3_ and *bla*_KPC−92_ were encoding on a Tn*4410* copy (~ 10 Kb) located on an IncFIB/IncFII multi-replicon plasmid (~ 110–115 Kb) along with *bla*_TEM−1 A_ and a truncated *bla*_OXA−9_ (W112X) (Figure S3). This plasmid shared a 95%-98% of coverage and 99.7–99.9% of identity with the pKpQIL-307 (116,499 bp; KY271403) of the ST307-Kp clone described by Villa *et al*.^5^. On the other hand, *bla*_CTX−M−15_ was found flanked by IS*26*, on a larger IncFIB plasmid (~ 175–239 Kb) with other antimicrobial resistance genes [*aac(3)-IIa*, *aph(3’’)-Ib*, *aph(6)-*Id, *qnrB1*,* sul2*,* bla*_OXA−1_ and *bla*_TEM−1B_] (Figure S4). This pCTX-M-15 plasmid shared a 77%-97% of coverage and 99.9% of identity with the previously described pKPN3-307_typeA detected in the ST307 clone (227,989 pb; KY271404)^5^.

## Discussion

In this study, we simultaneously detected KPC-Kp isolates belonging to the ST307 clone and resistant to ceftazidime-avibactam in ​​infected and/or colonized patients and in environmental samples from the ICU of our hospital during the early period of COVID-19 pandemic. The HiRC ST307 has been largely associated with nosocomial dissemination and outbreaks in hospitals and long-term care centers, showing a high capacity to acquire different antimicrobial genes, including KPC^5,6,11^. In our hospital, ST307 increased dramatically in 2018 and since then it has been mainly associated with KPC-3 between 2019 and 2020, but also with the OXA-48 enzyme in 2021 and 2020 ^11^.

Hospital surfaces and sinks in patient rooms, surgical wards and ICUs seems to play a critical role in the transmission of multi-drug resistant (MDR) organisms^15–17^. A study performed in our hospital demonstrated the persistence and evolution of the CTX-M-15-ST307-Kp clone, associated or not with KPC and OXA-48 production, in the patient care environment of our UCI from 2019 to 2023 ^18^. The presence of other MDR/ extremely-drug resistant (XDR) *K. pneumoniae* HiRCs (ST405, ST11) have also been described in the ICUs environment^18,19^. Long-term survival of *K. pneumoniae* in ICU environmental reservoirs could facilitate the horizontal gene transfer and further amplification of genes conferring resistance to first line antibiotics such as those encoding ESBLs and/or carbapenemases^20,21^. Our genomic analysis confirmed identical resistance genes in both clinical and environmental KPC-Kp isolates. Moreover, the sequences of the *bla*_KPC−3_/*bla*_KPC−92_- and *bla*_CTXM−15_-encoding plasmids described in this study were almost identical among them and closely related to those previously described by Villa *et al*.^5^.

A close genetic relationship was also observed by the cgMLST analysis (≤ 10 alleles) between all clinical and KPC-ST307-Kp sink isolates, highlighting the critical role that the hospital water system might play in the persistence and dissemination of this clone within our hospital. However, although the phylogenetic analysis of the KPC-92-ST307-Kp isolates supported possible transmission between patients and the environment, the directionality of spread is difficult to establish. In this sense, insufficient infection control measures and/or inappropriate patient handling during the COVID-19 pandemic could have contributed to a high cross-transmission of these isolates between both patients and sinks.

Recent research has emphasized the importance of effective cleaning protocols (rigorous hand hygiene and disinfection and regular monitoring of high-risk surfaces) and the need to reinforce strategies for reservoir identification to reduce the risk of MDR infection^22–24^. However, in 2020, the COVID-19 pandemic further exacerbated the challenges in managing MDR infections, particularly in ICU patients^25^. A previous study in our hospital showed that critically ill patients with COVID-19 were at an increased risk of infection with CPE and were associated with a higher mortality rate. KPC-Kp was the MDR bacteria most frequently isolated in these patients, reflecting the epidemiological situation in our hospital between 2019 and 2020 ^[11,13]^. In the present study, up to seven patients colonized or infected with ceftazidime-avibactam resistant KPC-ST307-Kp, had COVID-19 during their stay in the ICU.

The marked increase in ceftazidime-avibactam use in 2020 (1.41-fold compared to 2019) may have contributed to the selection and emergence of resistant KPC-Kp isolates in the ICU. Note that ceftazidime-avibactam was the only novel antimicrobial available during the COVID-19 pandemic in our institution for treating CPE infections. In this context, ceftazidime-avibactam was the most frequently used to prevent and treat superinfections, primarily caused by the KPC-ST307-Kp clone, in critically ill patients with COVID-19 admitted to our hospital (33% of cases in monotherapy and in 25% of cases in combination with other antimicrobials)^13^. Our results support that ceftazidime-avibactam resistance likely emerged through in vivo evolution primarily driven by the selective pressure of ceftazidime-avibactam therapy, rather than by a horizontal gene transfer event such as plasmid transmission. This interpretation is further reinforced by the mutational analysis of isolates harboring KPC-92 and by our previous studies with other KPC variants^9,26^.

The development of resistance in patients receiving ceftazidime-avibactam treatment underscores the complexity of managing infections caused by KPC-Kp and has forced the need for therapeutic alternatives as other novel combinations. In addition, the collateral sensitivity to carbapenems observed in ceftazidime-avibactam-resistant KPC-Kp strains has also raised the possibility of reconsidering carbapenems as a therapeutic strategy^27,28^. Nevertheless, high-level of carbapenem resistance and co-resistance to other novel antimicrobials has also been reported associated with KPC-Kp mutants or increased *bla*_KPC_ copy number usually in combination with porin loss^14,29–33^. In fact, we detected resistance to cefiderocol and cefepime-taniborbactam in patients and sink isolates that combined mutated KPCs with defects in membrane proteins. Despite increased MICs, aztreonam-avibactam, meropenem-vaborbactam, and imipenem-relebactam showed retained susceptibility, supporting their potential role as effective treatment options. Our results suggest that the high use of ceftazidime-avibactam during this period could have selected isolates already accumulating different resistance mechanisms leading to decreased susceptibility or even cross-resistance to last-line antibiotics in clinical isolates. However, we cannot elucidate which was the effect of the high use of ceftazidime-avibactam in patients may have had on the bacteria that are part of the hospital environment.

In addition, at the end of 2020 we also detected an outbreak of KPC-62-ST307-Kp resistant to both ceftazidime-avibactam and cefiderocol in four ICU patients that were not treated with these antimicrobials^14^. The overburdening of health resources during the COVID-19 pandemic and the ability of *K. pneumoniae* to survive in the patient care environment for prolonged periods may have contributed to the outbreak^34,35^.

In conclusion, our study highlights the high transmissibility of KPC-ST307-*K. pneumoniae* in the ICU of our Hospital during the COVID-19 pandemic, with the emergence of resistance to ceftazidime-avibactam and a major role of the ICU sinks as environmental reservoir(s). Our findings show that to understand the epidemiology and resistance dynamics of *K. pneumoniae* HiRCs in healthcare settings, and particularly within the ICUs, is critical to analyze with both microbiological and genomic studies the role of the environment aligned with continuous surveillance of both patients and environmental reservoirs to manage the threat posed by KPC-producing *K. pneumoniae* resistant to novel antimicrobials.

## Materials and methods

### Collection of samples and study design

 The study included all carbapenemase-producing *K. pneumoniae* isolates detected in clinical or surveillance samples from patients admitted at the ICU of Hospital Universitario Ramón y Cajal during 2020. These samples were collected as part of the routine of the clinical Microbiology Department. Rectal swabs were directly seeded on ChromID-ESBL and ChromID CARBA SMART biplate selective chromogenic agar plates (bioMérieux, Marcy-l’Etoile, France) and incubated at 37 °C for 24/48 h. Clinical isolates were recovered and cultured following established protocols. Patient’s data (demographic data, length of stay, antimicrobial therapy, microbiological samples, and date of sampling) were collected from the electronic medical records. This study was conducted according to the guidelines of the Declaration of Helsinki and was approved by the Hospital Universitario Ramón y Cajal Ethics Committee (reference 293/19). All experiments were performed in accordance with relevant guidelines and regulations. Patient consent was waived because it was not required by the Ethics Committee.

To perform a comparative analysis, a subset of 350 environmental samples from the patient care environment (sink, drain, siphon) collected between July and September 2020 in the same ICU as a part of the MicroCarbaFlux project (Ref. CC23140547) was included in the study^15^. Samples were collected with standard sterile swabs and aspiration probes, plated onto BD CHROMagar Orientation Medium, BD CHROMagar ESBL-Biplate, and mSuperCARBA (Becton Dickinson, Franklin Lakes, USA) and incubated at 37 °C for 24/48 h. Colonies of different morphotypes (size, color, and shape) were subcultured onto BD CHROMagar Orientation Medium and incubated for 24 h at 37 °C.

Bacterial identification was performed by MALDI-TOF-MS (Bruker-Daltonics, DE) in isolates from both patient and environmental samples.

### Infection control measures and intervention strategies

 Following the regional guidelines, infection control measures, including contact isolation and standard precautions, are implemented after each case of CPE detection^36^. Our facility’s protocol includes using a 4% chlorhexidine soap for patients’ daily hygiene, emphasizing hand hygiene of healthcare workers and visitors with an alcohol-based hand rub solution, using dedicated or disposable patient-care equipment and decontamination of the patients’ environment twice daily, focusing on frequently touched surfaces and equipment in the immediate vicinity of the patient. If equipment must be used with another patient, it is cleaned and disinfected according to its risk level (non-critical or semi-critical) with a disinfectant containing benzalkonium chloride and didecyldimethylammonium or with a 2% peracetic acid solution. For daily surface cleaning and disinfection, a combined detergent–chlorine solution is used. The standard ICU infection prevention protocol for decontamination regimens was also applied in all patients requiring orotracheal intubation as previously described^14^.

### Antimicrobial susceptibility testing

Standard broth microdilution (Sensititre-EUMDROXF) and disk diffusion method were used to antimicrobial susceptibility testing in clinical and environmental isolates, respectively^15^. Ceftazidime-avibactam resistant KPC-Kp isolates recovered from environmental samples were also tested by microdilution (Sensititre-EUMDROXF). Susceptibility testing results were interpreted using EUCAST-2025 criteria (https://www.eucast.org/clinical_breakpoints).

### Carbapenemase production

*K. pneumoniae* isolates collected from patients were phenotypically confirmed as KPC producers using the KPC/MBL/OXA-48 Confirm kit (Rosco Diagnostica, Taastrup, DE), the β-CARBA test (Bio-Rad, California, US), the O.K.N.V.I. RESIST-5 (CORIS BioConcept, Gembloux, BE), and the NG-Test CARBA 5 (Hardy Diagnostics, California, USA) immunochromatography tests. In isolates recovered from environmental samples, ESBL and carbapenemase production was screened by double-disk synergy test (DDST). KPC production was confirmed by multiplex PCR (*bla*_VIM−1_, *bla*_OXA−48_, *bla*_KPC_, *bla*_NDM_, and *bla*_GES_) and Sanger sequencing as previously described^37^.

### WGS and bioinformatics analysis

All KPC-Kp resistant to ceftazidime-avibactam from both patient and environmental samples were selected for the subsequent WGS analysis. KPC-3-Kp isolates recovered from patient samples before treatment with ceftazidime-avibactam were also included. Genomic DNA extraction was performed using the QIAamp DNA Mini Kit (Qiagen, GmbH, Hilden, Germany). Short-read sequencing was performed in all KPC-Kp isolates using Illumina NovaSeq 6000 and HiSeq4000 platforms (OGC, Oxford, UK). KPC-92-Kp and KPC-3-Kp isolates from patients 4 and 6 and the environmental KPC-92-Kp were also analysed using Oxford-Nanopore^®^ (MinION) technology. From these strains, complete hybrid assemblies were obtained from the combination of short- and long-reads using Unicycler, Flye and Pilon tools^38^.

Quality control and sequence processing was carried out as previously^39^. Genome assemblies were annotated by Prokka^40^. MLST was performed *in silico*. Acquired antimicrobial and disinfectant resistance genes were analysed (ResFinder, CGE database, http://genepi.food.dtu.dk/resfinder). Chromosomal point mutations were also studied using PointFinder^41^. Mutations in OmpK35 and OmpK36 porin proteins were confirmed using PCR and sanger sequencing. Mutations in genes previously associated with cefiderocol resistance in *K. pneumoniae* [siderophores (*fiu*), TonB-dependent energy transduction complex (*tonB*,* exbB*,* exbD*), PBP (*ftsI-pbp3*,* mdrA-pbp2*), enterobactin or ferrochrome receptors (*fhuA*,* fepA*), efflux transporter (*sugE*,* chrA*,* yicM-nepI*), other (*baeS/R*,* envZ-ompR*,* ompA*)] were also analyzed using Blast tool. A core genome MLST (cgMLST) analysis was also performed using the pipeline chewBBACA and the ST307-*K. pneumoniae* NCTN00000000 as the reference genome^42^. A minimum spanning tree was constructed and visualized using PHYLOViZ^43^. Variant calling analysis was performed with Snippy, and phylogenetic trees were obtained and constructed using iqtree2 and iTOL (https://itol.embl.de/) tools, respectively^44,45^. Mobile genetic elements and *bla*_KPC_ genetic context were characterized in the hybrid assemblies using PlasmidFinder, pMLST, Blast webtool and Proksee^46,47^.

Assemblies were deposited at DDBJ/ENA/GenBank under the project numbers: PRJNA741867, PRJNA985214, PRJNA747261 and PRJNA1205681.

## Supplementary Information

Below is the link to the electronic supplementary material.

Supplementary Material 1

Supplementary Material 2

## Data Availability

Sequence data were deposited at DDBJ/ENA/GenBank under the project numbers: PRJNA741867, PRJNA985214, PRJNA747261 and PRJNA1205681.
