# Supplementary material for: ICU environment as a reservoir of KPC-ST307-Klebsiella pneumoniae high-risk clone resistant to ceftazidime-avibactam
Source: Sci Rep. 2025 Aug 12;15:29486. doi: 10.1038/s41598-025-14987-w (PMC12343778; doi:10.1038/s41598-025-14987-w)
Supplement: Supplementary file 2 — Supplementary Material 2 [file 41598_2025_14987_MOESM2_ESM.docx]

**ICU environment as a reservoir of KPC-ST307-*Klebsiella pneumoniae* high-risk clone resistant to ceftazidime-avibactam**

**Marta Hernández-García^1,2*^, Marta Nieto-Torres^1^, Natalia Guerra-Pinto^1,2^, Juan Antonio Castillo-Polo^1^, Javier Saez de la Fuente^3^, Malkoa Michelena^1^, Manuel Ponce-Alonso^1,2^, Cruz Soriano-Cuesta^4^, Cristina Díaz-Agero^5^, Rafael Cantón^1,2*^, Teresa M. Coque^1,2^, Patricia Ruiz-Garbajosa^1,2^**

^1^Servicio de Microbiología, Hospital Ramón y Cajal and Instituto Ramón y Cajal de Investigación Sanitaria (IRYCIS), Madrid, Spain

^2^CIBER de Enfermedades Infecciosas (CIBERINFEC), Instituto Salud Carlos III (ISCIII), Madrid, Spain.

^3^Servicio de Farmacia, Hospital Universitario Ramón y Cajal and Instituto Ramón y Cajal de Investigación Sanitaria (IRYCIS), Madrid, Spain.

^4^Unidad de Cuidados Intensivos, Hospital Ramón y Cajal, Madrid, Spain

^5^Servicio de Medicina Preventiva y Salud Publica, Hospital Universitario Ramon y Cajal and Instituto Ramón y Cajal de Investigación Sanitaria (IRYCIS), Madrid, Spain

***Corresponding authors:** Rafael Cantón ([rafael.canton@salud.madrid.org](mailto:rafael.canton@salud.madrid.org)) and Marta Hernandez-García ([martahernandez1986@gmail.com](mailto:martahernandez1986@gmail.com)). Hospital Universitario Ramón y Cajal. Carretera de Colmenar Km 9,1. 28034-Madrid. Spain

**
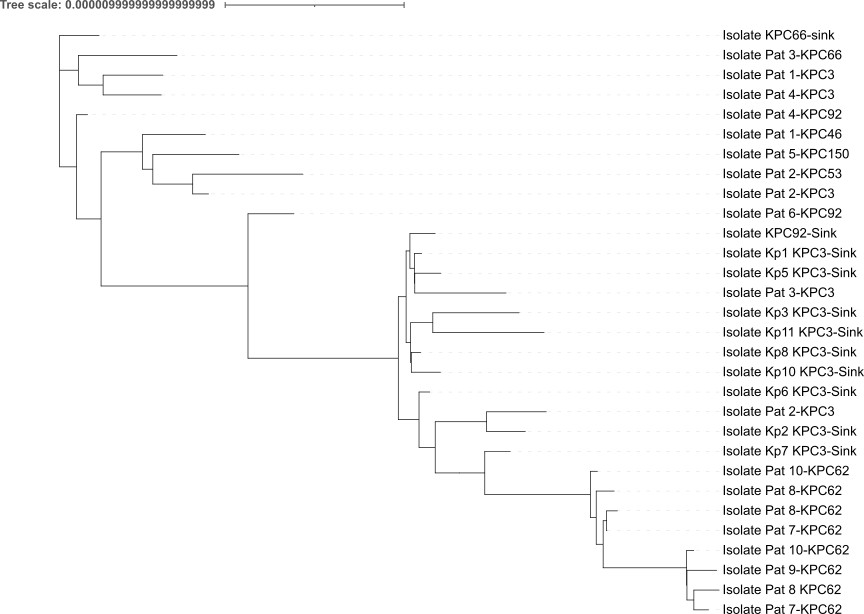
**

**Figure S1.** Core-genome maximum-likelihood phylogenetic tree constructed with all KPC-ST307-Kp isolates (ST307-*K. pneumoniae* NCTN00000000 was used as the reference genome).

**
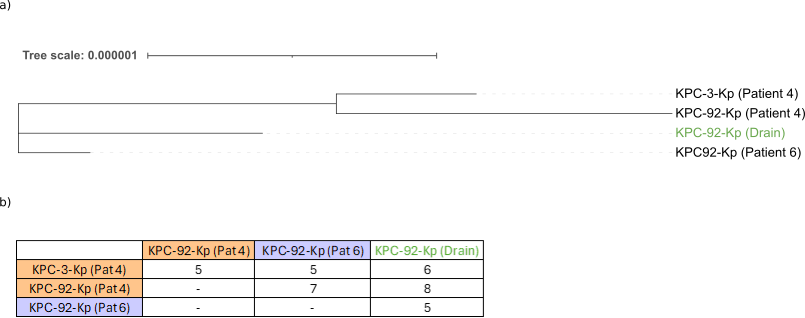
**

**Figure S2.** a) Core-genome maximum-likelihood phylogenetic tree constructed with the hybrid genome assemblies of the environmental KPC-92-Kp (used as the reference genome) and the clinical KPC-3- and KPC-92-Kp isolates from patients 4 and 6. b) Matrix table with the number of SNPs or INDELs for each pairwise comparison. The threshold number of SNPs defined for *K. pneumoniae* to be genetically related is ≤18 SNPs.

**
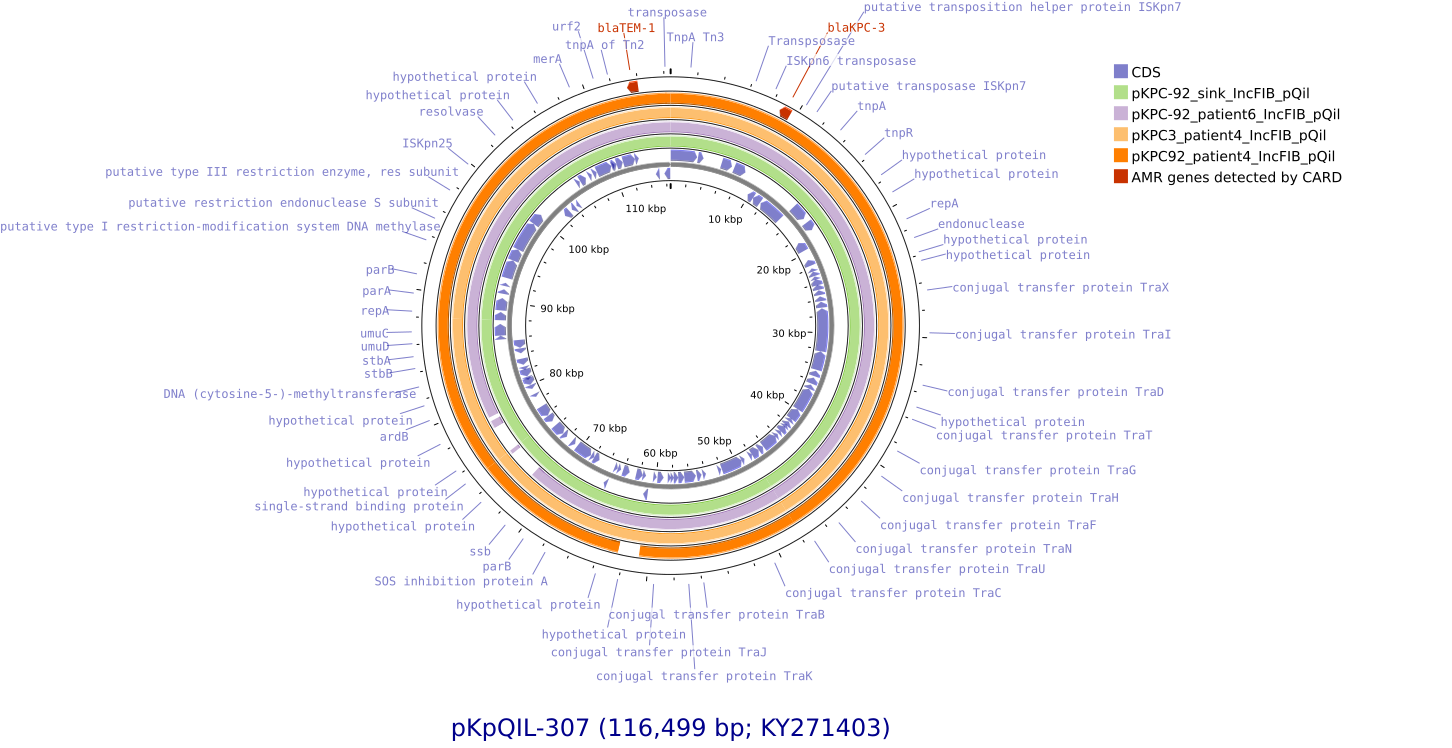
**

**Figure S3**. Reconstruction of the plasmid carrying *bla*_KPC-3_ or *bla*_KPC-92_ that was detected in the environmental KPC-92-Kp and the clinical KPC-3- and KPC-92-Kp isolates from patients 4 and 6. The pKpQIL-307 plasmid (accession number KY271403) previously described by Villa *et al* [5] was used as the reference plasmid.


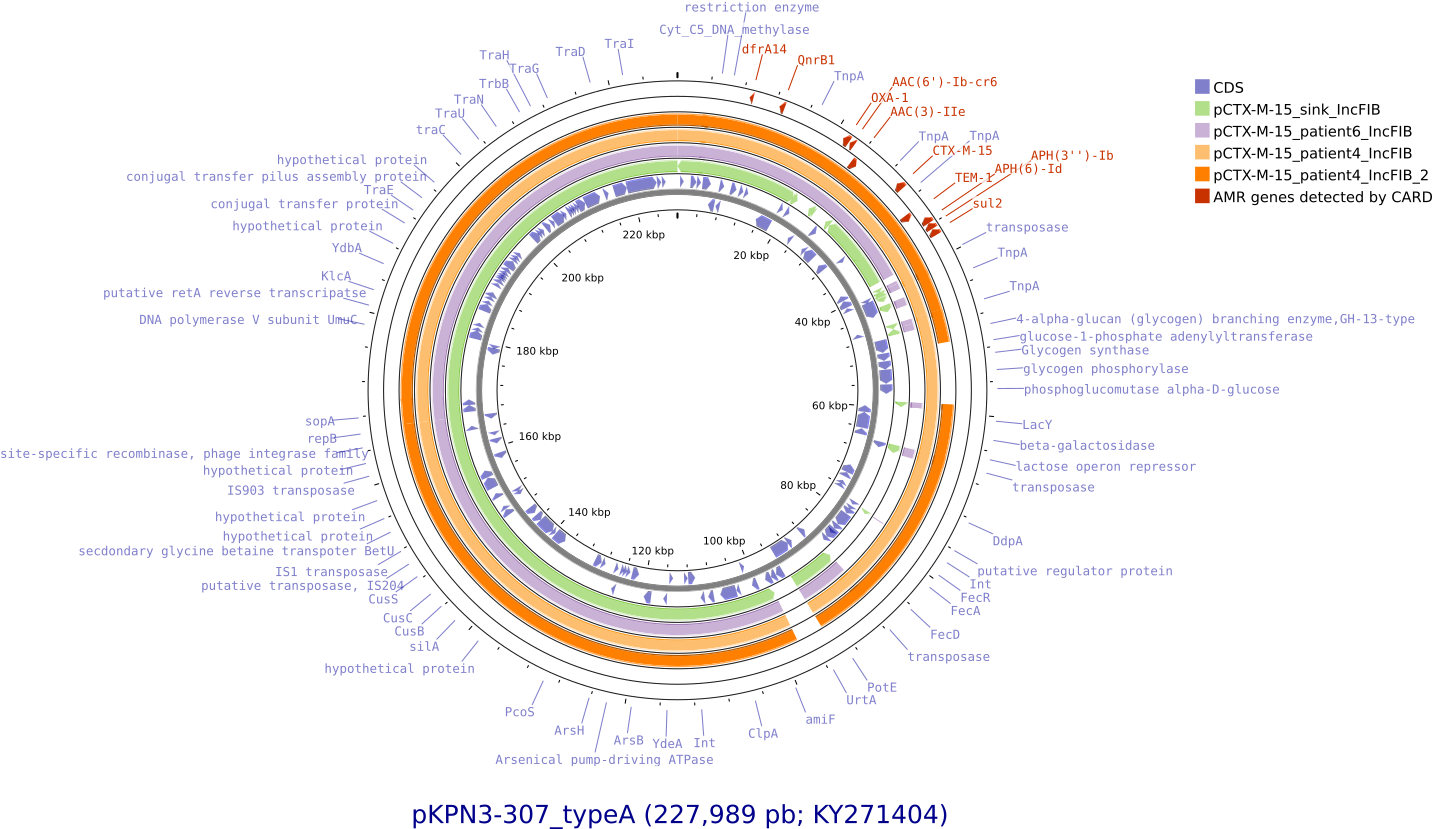


**Figure S4**. Reconstruction of the plasmid carrying *bla*_CTX-M-15_ that was detected in the environmental KPC-92-Kp and the clinical KPC-3- and KPC-92-Kp isolates from patients 4 and 6. The pKPN3-307_typeA plasmid (accession number KY271404) previously described by Villa *et al* [5] was used as the reference plasmid.
